# Efficacy of trastuzumab beyond progression in HER2 positive advanced gastric cancer: a multicenter prospective observational cohort study

**DOI:** 10.18632/oncotarget.10456

**Published:** 2016-07-07

**Authors:** Qian Li, Huiqin Jiang, Hong Li, Ruihua Xu, Lin Shen, Yiyi Yu, Yan Wang, Yuehong Cui, Wei Li, Shan Yu, Tianshu Liu

**Affiliations:** ^1^ Department of Medical Oncology, Zhongshan Hospital, Fudan University, Shanghai, China; ^2^ Department of Medical Oncology, Sun Yat-Sen University Cancer Center; State Key Laboratory of Oncology in South China, Guangzhou, China; ^3^ Department of Gastrointestinal Oncology, Key Laboratory of Carcinogenesis and Translational Research (Ministry of Education), Peking University Cancer Hospital and Institute, Beijing, China

**Keywords:** advanced gastric cancer, HER2, trastuzumab, treatment beyond progression

## Abstract

**Introduction:**

Trastuzumab plus chemotherapy is the standard first-line regimen in HER2 positive advanced gastric cancer (AGC), but lack of data in post-progression treatment. So, it is worth evaluating the efficacy of continuing trastuzumab after failure of the first-line trastuzumab based treatment.

**Methods:**

59 patients were enrolled from Zhongshan Hospital Fudan University, Sun Yat-sen University Cancer Center and Peking University Cancer Hospital between September 2012 and Oct 2015. Patients were divided into two groups according to the second line regimens: with or without trastuzumab. The primary endpoint was progression free survival of second line therapy (PFS2). Secondary end points included overall survival (OS), response rate, and adverse events (AEs).

**Results:**

Baseline factors were well balanced between two groups. 32 patients treated with trastuzumab plus second line chemotherapy (group A) and 27 patients received chemotherapy alone (group B). The median follow-up time was 7.60 months (range 1.50-32.50). Longer median PFS2 was observed in group A than in group B (3.1 vs 2.0 months, *P*=0.008). There was no significant differences of median OS2 calculating from the second line therapy (10.5 vs 6.5 months, *P*=0.172) between two groups. Response rate was 9.3% in group A compared with 3.7% in group B (*P*=0.617). AEs were similar in two groups including cardiac safety. Subgroup analysis showed that factors of male, age<65, good performance status, HER2 immunohistochemical (IHC) 2+ and poor response to first line indicated superior PFS2 in patients continuing trastuzumab to those treated with chemotherapy alone.

**Conclusion:**

Continuing treatment of trastuzumab beyond first line therapy progression showed effective and safe in AGC.

## INTRODUCTION

Gastric cancer is one of the leading causes of cancer-related death worldwide [1]. The incidence rate of gastric carcinoma is higher in Eastern Asia than that in the other part of the world, especially in China [2]. Most of Chinese patients have been diagnosed with unresectable or metastatic disease in their initial visit. The prognosis of advanced gastric cancer(AGC) is poor: median survival time is approximately 8-11 months [3–5]. Patients with human epidermal growth factor receptor-2(HER2) overexpression/amplification are accounted for 6.0%-29.5% in advanced gastric cancer [6–10]. Trastuzumab, a humanized monoclonal antibody that targets HER2, with chemotherapy is the standard treatment strategy for HER2 positive patients according to the result of ToGA trial [8]. In subgroup analysis of ToGA, median overall survival(OS) in patients with immunohistochemistry (IHC) 3+ or IHC 2+ plus fluorescence in situ hybridization (FISH) +, is 16.0 versus 11.8 months in trastuzumab and chemotherapy group, respectively. It was more prominent than the whole patients in ToGA (median OS 13.8 versus 11.1 months). However, there is no consistent treatment recommendation for those who progressed during first line palliative treatment with trastuzumab plus chemotherapy.

Progression during therapy generally implies resistance to the therapy and leads to change to another treatment regimen previously. The resistant to cytotoxic drug is due to the result of genetic instability inherent in cancer. But it is not applicable for biologic agents [11]. Several studies have also shown favorable prognosis to maintenance molecular-targeted drugs after disease progression such as Bevacizumab in metastatic colorectal cancer [11, 12] and Trastuzumab in metastatic breast cancer [13, 14]. Although trastuzumab is safe and effective in second line therapy [15, 16], whether trastuzumab beyond progression improves the prognosis is still unclear. This observation study is designed to analyze the benefit and risk associated with continuing trastuzumab treatments after first line progression.

## RESULTS

### Patient characteristics

**Figure 1:**
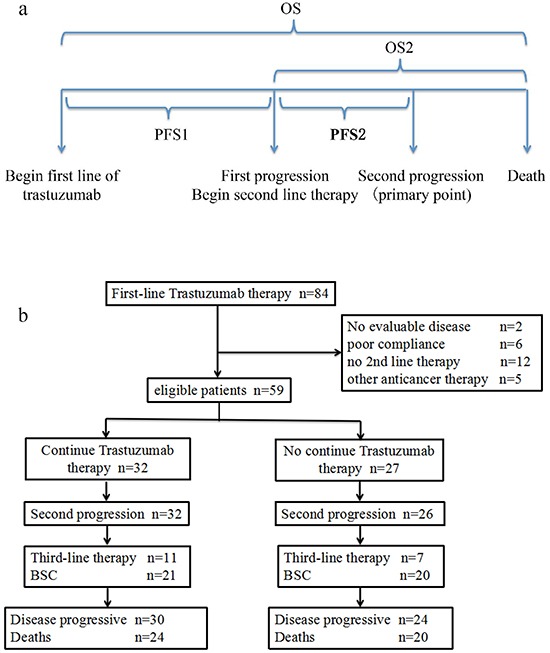
**A.** Schematic of patient observation periods **B.** disposition of patients (trial profile)

Between September 2012 and Oct 2015, 84 HER2 positive AGC patients who had disease progression during trastuzumab plus first line chemotherapy were registered in this study. 12 patients did not receive second line therapy. 6 patients did not received regular treatment or disease evaluation due to poor compliance. 2 patients had no evaluable disease according to RECIST 1.1. 5 patients were treated with other anticancer therapy. There were 59 eligible patients: 32 received trastuzumab beyond progression with second line chemotherapy; 27 received chemotherapy alone as second line therapy (Figure 1). Median PFS of first line therapy (PFS1) was 7.0months (95% CI 1.50-32.50), median trastuzumab cycle of first line was 8 (range 2-22) in total 59 patients. The baseline characteristics were well balanced in two groups including demographics, treatment profiles, clinical characteristics (Table 1).

**Table 1:** Baseline patient characteristics

	Group A (n=32)	Group B (n=27)	*P* value
Sex			0.063
Male	22(69%)	24(89%)	
Female	10(31%)	3(11%)	
Age (years)			0.128
<65	17(55%)	20(74%)	
≥65	14(45%)	7(26%)	
Tumor location			0.933
GEJ	11(34%)	9(33%)	
Other stomach	21(66%)	18(67%)	
Lauren			0.867
Intestinal	14(44%)	10(37%)	
Non-intestinal	5(16%)	5(19%)	
Unknown	13(40%)	12(44%)	
HER2 status			0.962
IHC3+	19(59%)	16(60%)	
IHC2+/FISH+	13(41%)	11(40%)	
Number of metastatic organs			0.432
<3	23(72%)	21(81%)	
≥3	9(28%)	5(19%)	
Metastatic site			
liver	20(63%)	15(56%)	0.589
Peritoneum	7(22%)	9(33%)	0.324
Lymph node	23(72%)	16(59%)	0.308
ECOG			0.315
0/1	29(91%)	21(78%)	
2	3(9%)	6(22%)	
First-line chemotherapy			0.127
Platinum based	17(56%)	9(33%)	
Non-platinum based	15(44%)	18(67%)	
First-line response			0.614
CR/PR	21(66%)	16(59%)	
SD/PD	11(34%)	11(41%)	
Second-line chemotherapy			
Fluoropyrimidine based	18(56%)	19(70%)	0.264
Platinum based	11(34%)	8(30%)	0.698
Irinotecan based	5(19%)	5(15.6)	0.518
Taxane/Docetaxel based	15(47%)	13(48%)	0.922
Third-line therapy			0.483
No	21(66%)	20(74%)	
Yes	11(34%)	7(26%)	
mTT1 (range, cycles)	7.5(2-22)	9.0(2-22)	0.577*
mPFS1 (95%CI, months)	9.00 (range 6.25-11.75)	6.60 (range 5.89-7.31)	0.134^#^

* Mann-Whitney U test,

# log-rank test

Abbreviation: GEJ, gastric esophagus junction; ECOG, European Cooperative Oncology Group; HER2, human epidermal growth factor receptor 2; IHC, immunohistochemistry; CR, complete response; PR, partial response; SD, stable disease; PD, progression disease; mPFS1, median progress free survival of first line therapy; mTT1, median cycles of first line trastuzumab therapy.

### Survival and efficacy

**Figure 2:**
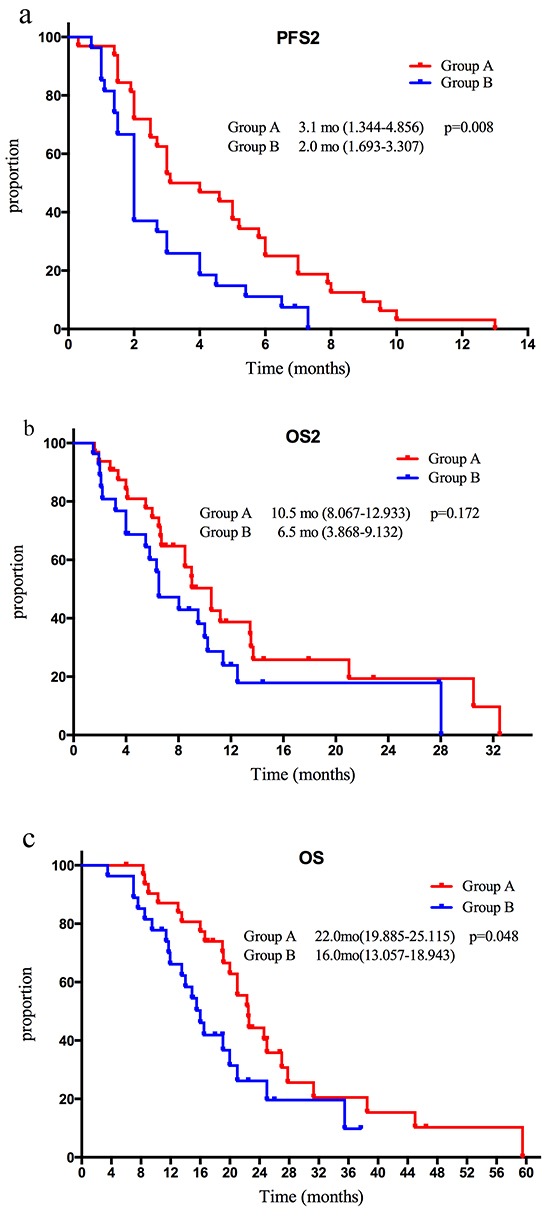
**A.** Kaplan-Meier curves for median PFS2 in two groups **B.** Kaplan-Meier curves for median OS2 in two groups **C.** Kaplan-Meier curves for median OS in two groups.

The median number of chemotherapy cycles plus trastuzumab cycles were 4 (range 1-15) in group A. Median chemotherapy cycles were 3(range 1-3) in group B. Median follow-up time was 7.60 months (range 1.50-32.50) for all enrolled patients, 6.33 months (range 1.50-28.03) for group A and 8.75 months (range 1.60-32.50) for group B, respectively. 58 patients had disease progression during second line therapy and 44 patients died of tumor until the last follow-up date. All patients underwent response evaluation. Median PFS2 was superior in group A (3.1 months (95% CI 1.3-4.8) versus 2.0 (95% CI 1.7-3.3), *P*=0.008). OS2 showed a trend of benefit in group A without statistical significance (10.5 months versus 6.5 months, *P*=0.172), while median OS was 22 months in group A compared with 16 months in group B, *P*=0.048 (Figure 2). There was no CR in either group. RR and DCR were 9.3% and 59.3% in group A (3 PR, 13 SD, 16 PD), and 3.7% and 33.3% in group B (1 PR, 8 SD, 18 PD). Neither RR nor DCR presented significant differences in two groups.

### Subgroup analysis

**Figure 3:**
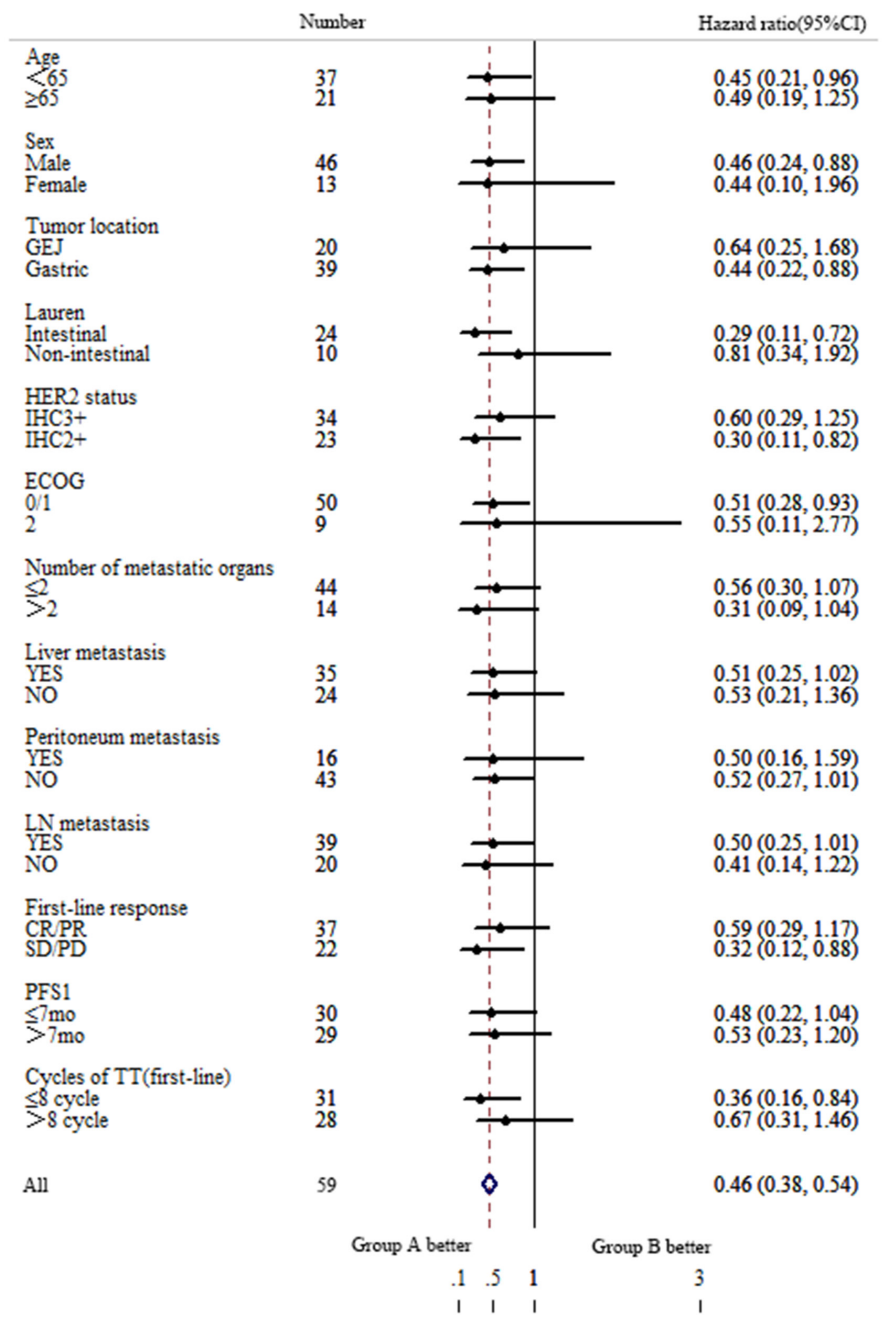
Forest plot of hazard ratios and 95% CIs for progress free survival of second line therapy assessed by subgroup factors ECOG, European Cooperative Oncology Group; HER2, human epidermal growth factor receptor 2; IHC, immumohistochemistry; CR, complete response; PR, partial response; SD, stable disease; PD, progression disease; mPFS1, median progress free survival of first line therapy; TT, trastuzumab.

The possible prognostic factors were all explored by univariate and multivariate analyses, including gender, age, location of primary tumor, baseline ECOG PS, Lauren type, number of metastatic organ, site of metastatic, radical surgery, HER2 expression, first line response, PFS of first line therapy and first line trastuzumab cycles Univariate analyses only showed significant differences in ECOG PS for PFS2. Good ECOG PS indicated less risk of progression (*P*=0.002). In patients who were male, age<65, ECOG PS≤1, underwent radical surgery, received less trastuzumab cycles in first line, PD/SD to the first line on PFS of second line therapy, there was a statistically significant difference between treatment groups in median PFS2 (Figure 3).

### Safety

The major incidences of grade 3-4 hematologic and non hematologic AEs for two groups were listed in Table 2. There was no significant difference between two groups including AEs. No patient had cardiac toxicity of any grade during the second line therapy including heart failure, arrhythmia and LVEF decreasing (defined as a ≥10% drop in LVEF to an absolute value <50%). There was no trastuzumab administration delay (delay > 1 week to the scheduled time) in group A. 2 patients in group A and 3 patients in group B had chemotherapy delay due to AEs. The number of patients who underwent a dose reduction was 1 and 2 in group A and B, respectively. There were no AE leading treatment discontinuation and treatment-related deaths in our study.

**Table 2:** Incidence of grade≥3 adverse events in two groups

Adverse events (≥3 grade)	Group A (N=32)	Group B (N=27)
Leucopenia	3	1
Neutropenia	4	3
Thrombocytopenia	3	0
Peripheral neuropathy	2	2
Nausea	1	0
Vomiting	0	0
Diarrhea	0	0
Fatigue	1	2
Anorexia	1	1
Liver dysfunction	0	0
Renal dysfunction	0	0
Heart failure	0	0
LVEF decreasing	0	0

## DISCUSSION

Our study showed that PFS2 of group A in which patients received trastuzumab beyond first line progression was significantly longer than that of patients with second line chemotherapy alone. To our knowledge, study about trastuzumab treatment beyond progression has never been reported previously in AGC. Actually, we noticed some physicians had already chosen this option [9] or suggested it as an alternative in clinical practice [10, 17–19]. However, there is few clinic data about second line anti-HER2 therapy, especially in those patients pre-treated with trastuzumab. According to the results of a randomized study of trastuzumab emtansine (T-DM1) versus taxane in patients with previously treated HER2-positive advanced gastric cancer (GATSBY) [20], T–DM1 did not show an efficacy benefit over taxane. There were 79.5% patients in arm T-DM1 with prior trastuzumab treatment and 76.3% in arm taxane. Median OS and PFS were 8.6 months and 2.9 months versus 7.9 months and 2.7months in each arm with no significant difference. In our study median PFS in trastuzumab beyond progression group was superior to chemotherapy (3.1 months versus 2.0 months), while OS2 was 10.5 months versus 6.5 months with no statistic difference. PFS of trastuzumab arm in our study is similar to GASTBY, which indicated the data in this observational study was credible.

TyTAN trial was another randomized phase III study for the anti-HER2 therapy in pretreated patients with HER2 positive advanced gastric cancer [21]. Lapatinib, a dual HER2 and epidermal growth factor receptor inhibitor, has failed to prove the efficacy in second line anti-HER2 therapy compared with chemotherapy. Subgroup analysis in both GASTBY and TyTAN trials also showed no benefit in those patients who had prior HER2-targeted therapy. Several possibilities may explain the differences. Firstly, the patients who continued trastuzumab treatment were a part of enrolled patients in GATSBY and TyTAN. Furthermore there was only 15 patients treated with trastuzumab previously in TyTAN [21]. Secondly, the primary end point was PFS in our study while OS in those two studies. So, the subgroup analysis was on OS but not PFS in theirs. PFS is an attractive end-points for clinical trials because they are available earlier than overall survival, not influenced by subsequent treatments, and needed relatively smaller study sample size. Therefore we chose PFS as our primary endpoint. Data of OS were also analyzed in details as a second endpoint. OS2 in group B was 6.5 months which is similar to the reported average for second line chemotherapy trials [22]. There was a trend of long lives (from second line to death) for patients continuing trastuzumab beyond progression, but without significant difference between two groups. Therefore, another analysis was performed in our study: trastuzumab treatment beyond progression was favor on overall survival calculated from the first line therapy. In Shitara's study [9], OS of 43 HER2 positive patients treated with trastuzumab was 24.7 months. Notably, 22 patients continued with trastuzumab beyond progression. Continuing trastuzumab and long exposure to trastuzumab may explain the long OS compared with the results of a subset analysis among those patients with IHC 3+ or IHC 2+ plus FISH + in TOGA [9]. Median OS of group A in our study appeared to be close to Shitara's (22.0months vs. 24.7 months) while group B was consistent with the results of TOGA (16.0 months vs. 16.0months). Above all, it suggested that our results are robust.

In subgroup analysis, there were several unexpected results. Firstly, patients with HER2 IHC2+ puls FISH+ appeared to be benefit more from trastuzumab treatment beyond progression. Median PFS2 was 3.0 months in group A while 1.5 months in group B for patients with HER2 IHC2+ puls FISH+. HER2 3+ usually indicated long survival in patients treated with trastuzumab than those patients with HER2 IHC ≤ 2+ [8, 23, 24]. A higher ratio of HER2/CEP17 or HER2 gene copy number were also associated with better outcomes in patients with IHC ≤ 2+ [23]. Actually patients with IHC 3+ also showed a superior trend of PFS2 to IHC 2+ in our study: 3.0 months vs 2.0 months in all enrolled 59 patients while4.6 months vs 3.0 months in group A. So it did not conflict with previous reports. Secondly, patients experienced less trastuzumab cycles were in favor from continuing trastuzumab treatment. Published data indicated that long duration of exposure to trastuzumab would reduce the risk of death in advanced gastric cancer [9, 16]. So we assume that patients progressed rapidly may benefit from retaining trastuzumab due to insufficient anti-HER2 therapy. Thirdly, patients whose best response to the first line therapy was SD/PD indicated a statistically significant PFS benefit in maintaining trastuzumab following progression. In our study, PD, SD, PR and CR of first line therapy was 5, 17, 35 and 2 patients, respectively. The risk of progression was lower with trastuzumab plus chemotherapy versus chemotherapy alone (*P*=0.027, HR 0.265) in further analysis to those SD patients. No obvious difference was observed in patients with PR. Statistical analysis could not be assessed in patients of PD or CR due to small sample size. Furthermore, subgroup analysis on OS2 was carried out. However, HER2 status, trastuzumab cycles of first line and response to first line were not prognostic factors. So it was warrant further analysis to identify the population who might benefit more from continuing trastuzumab beyond progression.

There are always limitations for observational cohort study based largely on the factor that patients are not randomly assigned to each group being compared. Furthermore the explanatory analyses of subgroups of patients were post hoc, but not pre-planned. At last, small sample size is also weak point in our study. Thus, a randomized prospective study is warranted. However, low positive rate of HER2, short survival after progression and that few patients were eligible for second line therapy, make it difficult to recruit patients and therefore, even more difficult to do randomized study.

In conclusion, we found that maintaining trastuzumab beyond first line therapy progression improved the prognosis of HER2 positive AGC patients. The results support the treatment model in other cancer: progression may not indicate a loss of clinical benefit from trustuzmab. All in all, continuous administration of trastuzumab is an effective and safe clinical practice.

## PATIENTS AND MEHODS

### Patients

All patients in this study were from Zhongshan hospital, Fudan University, Cancer Center of Sun Yat-sen University and Peking University cancer hospital. During September 2012 and Oct 2015, we prospectively collected HER2 positive AGC patients who received first line palliative treatment including trastuzumab. HER2 status was detected locally in these three centers. Due to the subset analysis of ToGA trial patients, HER2 positive was defined as IHC 3+ or IHC 2+ plus FISH positive in this study. The additional principal inclusion criteria were as follows: (1) pathology and medical imageology proven inoperable advanced gastric adenocarcinoma; (2) received trastuzumab plus chemotherapy as the first-line palliative chemotherapy and trastuzumab treatment duration was more than 6 weeks; (3) with measurable lesion with a diameter 20 mm using conventional computed tomography (CT) or magnetic resonance imaging (MRI) scans or 10 mm using spiral CT scans; (4) CT or MRI confirmed disease progression during first line therapy; (5) patients begin second line therapy within 8 weeks of the last dosage of first line trastuzumab; (6) Eastern Cooperative Oncology Group performance status (ECOG PS) of 0-2; (7) left ventricular ejection fraction(LVEF) more than 50 percents; (8) sufficient bone marrow, liver and renal function. The main exclusion criteria included: (1) switch to second line chemotherapy due to toxicity; (2) second line chemotherapy regimen is as same as first line or a part of first line chemotherapy; (3) treatment with any other anticancer therapy (lapatinib, immunotherapy, etc); (4) >1 prior line of therapy for AGC; (5) history of intolerance reaction or hypersensitivity to trastuzumab; (6) Grade ≥2 adverse event except alopecia.

### Treatment

Trastuzumab was administrated every 3 week with a dose of 6mg/kg (8mg/kg for first dose if second line therapy began more than 3 weeks from the previously administration). There were no protocol-specified chemotherapy regimens. All the patients were divided into two groups according to physician's decision: (1) group A, continuing trastuzumab beyond progression with second line chemotherapy; (2) group B, second line chemotherapy alone. Dosage adjustment or treatment delay was also made by physicians as usual clinical practice when toxicity occurred.

### Follow-up

Clinical and pathological characteristics, first line treatment information were recorded at baseline, while toxicity according to version 4.0 of Common Terminology Criteria for Adverse Events (AEs) was recorded every cycle. Patients received CT/MRI for response evaluation every 8 weeks according to Response Evaluation Criteria in Solid Tumors (RECIST)(version 1.1) or earlier if there are indications of treatment failure. Left ventricular ejection fraction (LVEF) was assessed at baseline and at least every 12 weeks. Patients were considered on study until death or loss to follow-up. The last date of follow-up was January 31th, 2016.

### Statistical analysis

The primary endpoint of this study was PFS of second line therapy (PFS2) defined as the time from the start of second line therapy to the date of second line therapy progression or the date of tumor related death. The second endpoints were OS defined as time from the start of second line therapy to death from any cause (OS2), overall survival from the beginning of first line therapy to death (OS), response rate (RR) defined as complete response (CR) plus partial response (PR), disease control rate (DCR) defined as CR plus PR plus stable disease (SD) and cardiac toxicity.

OS and PFS curves were estimated with the Kaplan-Meier method. Baseline characteristics, RR, DCR and adverse events were calculated with Chi-squared tests or with Fisher's exact test, for homogeneity or for trend. Variables showing a trend for association with PFS and variables that were known to have prognostic value were selected to evaluate by univariate and multivariate analyses using a Cox proportional hazards model. Datas were presented as an HR and a 95% CI. Stratified log-rank tests and Cox regression analyses were also carried out to find out the differences in subgroups. Statistical analyses were carried out using SPSS 19.0 software (IBM SPSS Statistics, Chicago, IL, USA). All tests were two-sided, and *P* <0.05 was considered statistically significant.

### Ethics statement

All patients signed written informed consent for their information to be used for study. This study was approved by the Research ethics committees of Zhong shan hospital, Fudan University, Cancer Center of Sun Yat-Sen University and Peking University cancer hospital.
